# Cross-country comparisons of trends in adolescent psychosomatic symptoms – a Rasch analysis of HBSC data from four Nordic countries

**DOI:** 10.1186/s12955-019-1097-x

**Published:** 2019-02-06

**Authors:** Curt Hagquist, Pernille Due, Torbjørn Torsheim, Raili Välimaa

**Affiliations:** 1 Centre for Research on Child and Adolescent Mental Health, Karlstad University, SE-651 88 Karlstad, Sweden; 2 National Institute of Public Health, University of Southern Denmark, DK-1353 Copenhagen K, Denmark; 3 Department of Psychosocial Science, University of Bergen, NO-5020 Bergen, Norway; 4 Department of Health Sciences, University of Jyväskylä, FI-40014 Jyväskylä, Finland

**Keywords:** Adolescents, HBSC, Psychosomatic symptoms, Rasch measurement theory, Trend analyses, Differential item functioning

## Abstract

**Background:**

To analyse the psychometric properties of the HBSC Symptom Checklist (HBSC-SCL) on psychosomatic symptoms with a focus on the operating characteristics of the items, and on the impacts of measurement distortions on the comparisons of person measures across time and between countries.

**Methods:**

Data were collected in 1993/94, 1997/98, 2001/02, 2005/06, 2008/09, 2013/14 in Denmark, Finland, Norway and Sweden as part of the Health Behaviour in School-aged Children (HBSC) study. Data comprised 116,531 students 11, 13 and 15 years old. Rasch analysis was conducted of the HBSC-SCL consisting of eight items with a focus on Differential Item Functioning (DIF) and item threshold ordering. The impacts of DIF and threshold disordering on trend analyses were analysed in a subsample consisting of 15 years old students.

**Results:**

One item shows evidence of severe DIF and the categorisation of some items does not seem to work as intended. Analyses of changes based on proportions of psychosomatic symptoms show that bad item functioning affects some comparisons between countries across time: A four percentage point difference between 15 years old girls in Finland and Sweden concerning the rate of increase of psychosomatic symptoms from 1994 to 2014 disappears when the problems with DIF and disordered item thresholds are taken into account. Although the proportions of students with psychosomatic symptoms are clearly higher 2014 than 1994 in all four countries the shape of most trends is nonlinear.

**Conclusions:**

Some of the cross-country comparisons were distorted because of DIF and problems related to disordering of the item thresholds. The comparisons among girls between Finland and Sweden were affected by the problems pertaining to the original measure of psychosomatic symptoms, while the trend patterns among boys were not much affected. In addition to confirming increasing rates of adolescent mental health problems in the Nordic countries, the substantive analyses in the current study show that Finland is joining Sweden in having the sharpest increase among older adolescents, in particular among girls.

To improve the functioning of the scale the DIF item could be removed or replaced and response categories collapsed in post hoc analyses.

## Background

In the wake of growing and worldwide concerns about deteriorating child and adolescent health, especially mental health, studies on time trends and comparisons between different countries are drawing great interest [1]. An international systematic review published a few years ago showed increasing mental health problems in many Western countries, most consistently for emotional problems, in particular among girls [2]. Another recent review [3] showed that there have been periods of increase as well as decrease in symptom prevalence in emotional problems and antisocial behavior in high-income countries. Among European countries, there are no general trend patterns of young people’s mental health [4, 5]. The complexity of the trend patterns is confirmed by trend analyses of adolescent mental health based on data from the Health-Behaviour in School-aged Children (HBSC) study for the 1994–2010 time period. The results conveyed different shapes of the trends [6]. These ambiguous trend patterns were also confirmed by a recent systematic literature review and meta-analysis of adolescent psychosomatic health complaints [7]. Only for Northern Europe there was a significant increasing trend.

The views of adolescent mental health frequently conveyed in some countries have been questioned for conceptual and methodological reasons. Objections have been raised concerning the validity of the measures, in particular because most studies are self-reports focusing on symptoms, ignoring the consequences for every-day life [8]. Also, concerns have been raised about the psychometric properties of the measurements used with respect to their invariant properties, i.e. whether the measures enable proper comparisons across time as well as among different socio-demographic groups [9].

Measures of mental health may work differently across countries because of mistranslations, cultural differences or other reasons. Similarly, measures may work differently across time because of changes in attitudes or conceptualisations. In a recent paper based on Finnish HBSC-data for the 1994–2014 time periods, in particular the item feeling depressed showed evidence of Differential Item Functioning (DIF) across time [9]. Previously, only a few analyses of DIF have been conducted on the HBSC-SCL. An analysis of data from year 2005 including 41 countries revealed that one of the eight items showed evidence of cross-country DIF, which was the item concerning sleeping difficulties [10].

In the present paper we address challenges in cross-country comparisons of adolescent mental health trends using data from four Nordic countries (Denmark, Finland, Norway, Sweden) belonging to the same geographical region, all being social welfare states.

The purpose of the present study is to analyse the psychometric properties of the HBSC-SCL using Rasch Measurement Theory with a focus on the operating characteristics of the items, as well as on the impacts of lack of measurement invariance on the comparisons of person measures across time and between countries.

## Methods

The study makes use of data collected in the HBSC study among students 11, 13 and 15 years old. The HBSC study is conducted in collaboration with the WHO Regional Office for Europe and it currently includes 48 countries and regions across Europe and North America. In the HBSC-study repeated data collections have taken place every fourth year since the 1980s. Data are collected in schools with a questionnaire which is completed anonymously in the classroom.

For the purpose of this study data from Denmark, Finland, Norway and Sweden are used, including data from six consequent surveys: 1993/94, 1997/98, 2001/02, 2005/06, 2008/09, 2013/14. The entire data set comprises a total of 116,531 students.

Rasch analysis was conducted of the HBSC-SCL which is a composite measure consisting of eight questions. In Table 1 the items are listed in English, Danish, Finnish, Norwegian and Swedish.

**Table 1:** Items on psychosomatic symptoms presented in English and four Nordic languages

In the last 6 months, how often have you had the following complaints?				
English	Finnish	Swedish	Norwegian	Danish
Headache	päänsärky	huvudvärk	hodepine	hovedpine
Stomach-ache	vatsakipu	ont i magen	vondt i magen	mavepine
Backache	selkäkipu	ont i ryggen	vondt i ryggen	ondt i ryggen
Feeling low	masentuneisuus (feeling depressed)	känt mig nere	følt deg. nedfor (trist)	været ked af det
Irritability or bad temper	ärtyisyys tai pahantuulisuus	varit irriterad eller på dåligt humör	*v*ært irritabel eller i dårlig humør	været irritabel/ i dårligt humør
Feeling nervous	hermostuneisuus	känt mig nervös	vært nervøs	været nervøs
Difficulties in getting to sleep	vaikeuksia päästä uneen	svårt att somna	vanskelig for å sovne	svært ved at falde i søvn
Feeling dizzy	huimauksen tunne	känt mig yr	vært svimmel	været svimmel

The response categories for all of these eight items are ‘About every day’, ‘More than once a week’, ‘About once a week’, ‘About once a month’ and ‘Seldom or never’. The categories are ordered in terms of implied frequency and the higher frequency, the higher the degree of psychosomatic symptoms.

### Analyses

The Rasch partial credit model for polytomous data [11] was used to examine the psychometric properties of the HBSC-SCL scale on psychosomatic symptoms, in particular if the instrument could be used for invariant comparisons between countries and across time. In the Rasch analysis the responses to each item were summarised and non-linearly transformed to a logit scale, which is common for both item and person location values [12]. While the location parameter is the only item parameter in the Rasch model for dichotomous data [13], in the Rasch model for polytomous data [14] there is an additional type of item parameter, the threshold parameter. The thresholds are partitioning the latent continuum of an item into ordered categories, one more than the number of thresholds [14]. The estimates of the thresholds need to be successively ordered. Disordered thresholds mean that the item categories do not work as intended [14]. Because the item thresholds appeared disordered in the current analyses, in the Rasch analysis two pairs of response categories (‘About every day’& ‘More than once a week’ and ‘About once a week’ & ‘About once a month’) were collapsed ending up with three response categories for each of the items [15].

In Rasch modelling the traditional data-model relationship is turned upside down. While misfit in statistical modelling is handled by inclusion of additional variables, the Rasch model is considered a formal representation of measurement against which data are examined [12]. Since the Rasch model has invariance as an integral property, misfit between the data and the model is an indication that the items do not work the same way for all individuals and groups of individuals in the sample. The items may work differently for persons at different locations along the latent variable as well as across different sample groups that are to be compared, i.e. there is evidence of DIF. In assessing invariance, the Expected Value Curve (EVC) is a useful graphical tool, complementing formal test statistics. In a polytomous item the slope of the EVC is a function of the distances between the item thresholds, i.e. the closer the thresholds are located, the steeper the slope.

The EVC predicts the responses to the items as a function of the items’ and persons’ locations on the latent trait. These expected values are compared with the observed values which should ideally fit perfectly with the EVC. There is no DIF if an item works in the same way for different sample groups, i.e. if members of all sample groups score the same on an item given the same location on the latent trait. In this case where there is no DIF, only one EVC is required. In contrast, if members of one sample group score higher on an item than members of another group given the same location on the latent trait, i.e. DIF is evident, one EVC for each sample group is required to predict the responses on an item. If the EVCs are parallel, the DIF is referred to as uniform; if the difference varies along the latent trait and the EVCs are non-parallel, the DIF is referred to as non-uniform [16]. Recent advances of the analysis of DIF has shown that real DIF has to be distinguished from artificial DIF. While real DIF affects person measurement, artificial DIF does not [16–18].

DIF was analysed through two-way analysis of variance (ANOVA) of standardised residuals. That procedure examines each item with respect to a class interval main effect, a group effect and a class interval by group interaction effect. The F-values give the rank order of DIF among the items [19]. While differences in item slope values between countries may be indicative of non-uniform DIF, disordered threshold values will make the slope steeper and may affect comparisons between countries. Therefore, not just cross-country DIF was analysed but also the possible impact of disordering of latent item thresholds. Because of the link between item thresholds and DIF, the threshold parameters were estimated twice, before and after the DIF item was removed, in order to examine if DIF was affected by disordered thresholds and vice versa.

Two formal tests of local independence were conducted: Analysis of the correlations between the person-item residuals, and principal component analysis (PCA) of the item residuals. In the PCA, the loadings directed the items into two subsets. The person location values generated from the two subsets of items were compared and the differences in person location values assessed for each person with independent t-tests.

The subsequent descriptive analyses were based on the person estimate values generated by the Rasch analysis. Because age specific analyses are preferred when reporting rates of young people’s health, and reporting all three age groups would have been too extensive, the analyses were confined to 15 years old boys and girls, i.e. the age group commonly being in the focus of reports on deteriorating adolescent mental health.

Four item sets differing with respect to number of response categories and number of items were analysed more closely and separately for boys and girls, including analyses of the impact of DIF and disordered thresholds on person measures used for cross-country comparisons, at the first (1994) and last (2014) year of investigation. The following item sets were analysed:

- Set A: the original set of 8 items with 5 response categories.
- Set B: a revised set of 8 items with 3 categories. Because of a big number of disordered thresholds in the original set, two pairs of categories (‘About every day’ & ‘More than once a week’ and ‘About once a week’ & ‘About once a month’) were collapsed.
- Set C: a revised set of 7 items with 5 categories. Item Feeling Low was removed.
- Set D: a revised set of 7 items with 3 categories. Item Feeling Low was removed and two pairs of categories (‘About every day’ & ‘More than once a week’ and ‘About once a week’ & ‘About once a month’) were collapsed.

Effect sizes of differences in mean values between countries at the first and last years of investigation were calculated using Cohen’s d.

In preparation of the descriptive trend analyses, the logit values from the Rasch analysis were rescaled in two ways in order to facilitate the interpretations of the results: a) the direction of the original variable was reversed implying that the higher the value on the transformed scale the worse health; b) the scale was transformed linearly to a score range between 0 and 100 where a score of 0 represents the lowest degree of psychosomatic problems and a score of 100 the highest, thereby avoiding negative values.

The trends in psychosomatic symptoms based on mean values across years of investigations were also displayed graphically, based on the original item set and the revised set consisting of 7 items and 3 response categories Similarly, trend analyses were conducted based on the proportion of students on or above the 90th percentile (i.e. higher degree of psychosomatic symptoms), at the first year of investigation for the entire sample among 15 years old boys and girls.

The Rasch analysis was performed with the software RUMM2030, which uses a pairwise conditional method of estimation for the item parameters in which person parameters are eliminated and the person estimates were obtained using a weighted likelihood method which reduces the bias in the person estimates taking the item parameters as known [20].

## Results

### Rasch-analysis of original set of eight items with five response categories

**Fig. 1:**
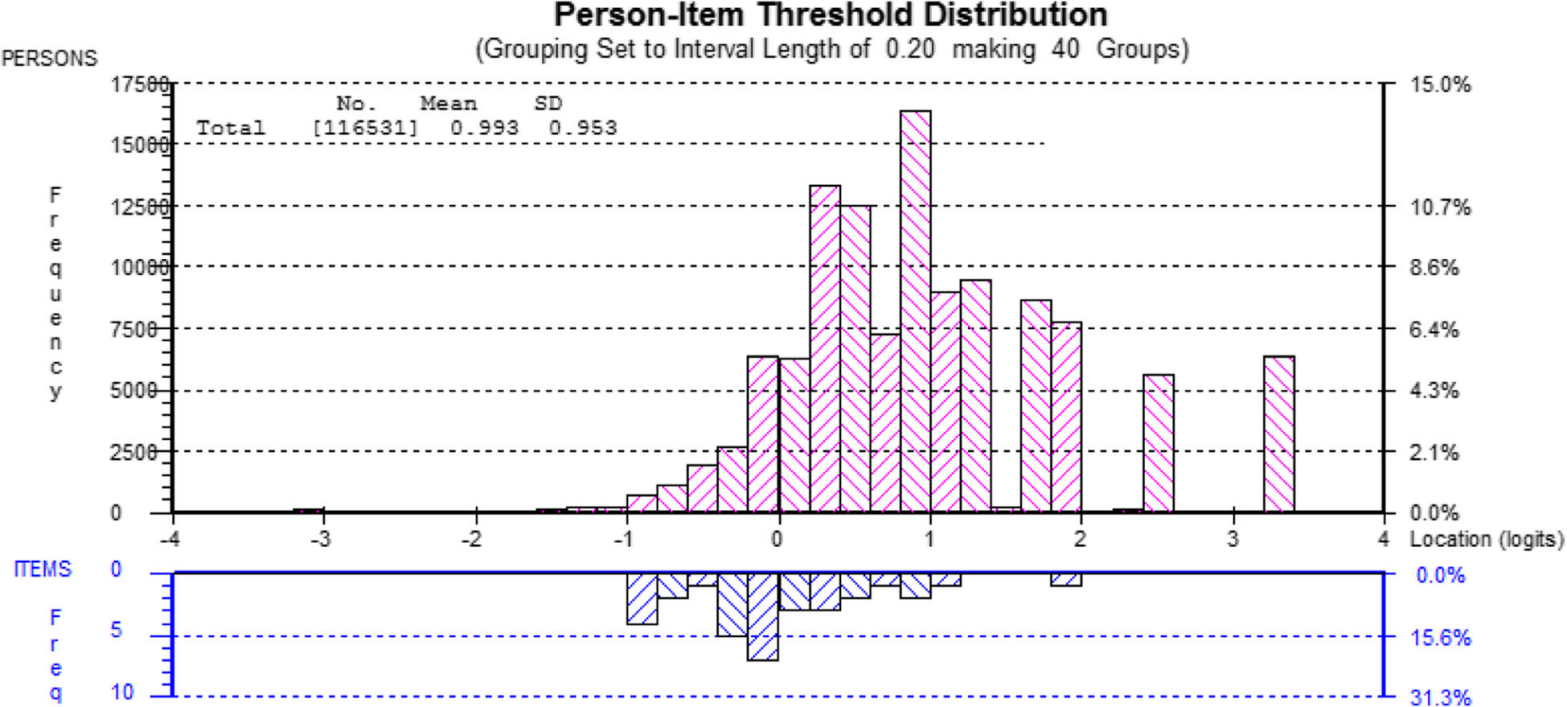
Person-item threshold distribution for the set of eight items, with five response categories. Entire sample of students 11, 13 and 15 years old. The higher the value, the less psychosomatic symptoms

Figure 1 shows the location of the item thresholds relative to the distribution of the persons for the original set of eight items with five response categories including all boys and girls 11, 13 and 15 years old.

Figure 1 shows that the locations of the persons are skewed to the left with a mean of 1, reflecting a population with a relatively good psychosomatic health. The item thresholds are dislocated relative to the persons and appear at the lower end of the variable, where persons with higher degree of psychosomatic symptoms are located.

**Fig. 2:**
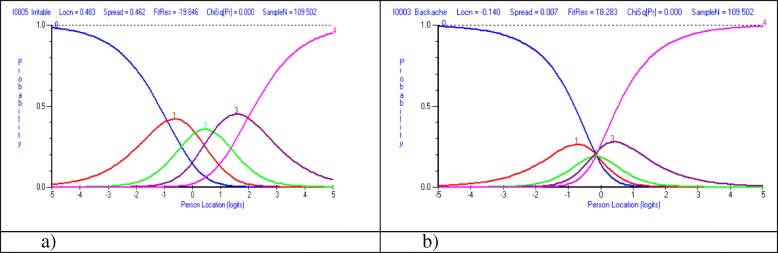
**a** Category probability curve for item “Have been irritable or in a bad temper” for set of eight items with five response categories. **b** Category probability curve for item “Backache” for set of eight items with five response categories. Entire sample of students 11, 13 and 15 years old

Figure 2 a-b shows the category probability curves for the items irritable/bad temper and backache for the set of eight items with five response categories including all boys and girls 11, 13 and 15 years old.

Figure 2 a shows an item where the estimates of the item threshold parameters that are defining the successive categories appear in the right order. High values on the variable mean that the probability of scoring a high value on item Irritable is high, and consequently, low values mean a low probability. Having a value in the centre of the continuum means a high probability responding in either of the two categories ‘More than once a week’(1) or ‘About once a week’ (2).

Figure 2 b shows a quite different pattern where the thresholds are disordered. For the item Backache regions of the continuum are undefined and the categories do not constitute increasing levels of the variable. A person who is located at around 0 logits is less likely to respond in category 2 (about once a week) than in the adjacent categories 1 (about every day), 3 (about once a month) and 4 (seldom or never).

In Table 2 item location and threshold values are shown for the set of eight items with five categories including all boys and girls 11, 13 and 15 years old.

**Table 2:** Item location and centralised threshold values for the set of eight items with five response categories. Entire sample of students 11, 13 and 15 years old. The lower the item location value, the more severe symptom

Item		Location	Threshold 1	Threshold 2	Threshold 3	Threshold 4
It 1	Headache	0.023415	−0.786540	− 0.161090	0.130786	0.816848
It 2	Stomach-ache	−0.230490	*− 0.749190*	*− 0.094010*	*−0.283000*	*1.126203*
It 3	Backache	−0.140470	*−0.023660*	*0.062261*	*−0.123770*	*0.085168*
It 4	Feeling low	−0.085780	−0.732560	0.000632	0.059639	0.672292
It 5	Irritability or bad temper	0.482598	−1.376570	−0.376840	0.260856	1.492549
It 6	Feeling nervous	0.025897	−1.003510	− 0.264530	0.253783	1.014259
It 7	Difficulties in getting to sleep	0.271704	*−0.242570*	*0.008973*	*0.143810*	*0.089783*
It 8	Feeling dizzy	−0.346880	*− 0.419110*	*0.244419*	*0.104895*	*0.069796*

Disordered thresholds marked in italics

Table 2 shows that disordered thresholds appear for four of the eight items. The items stomach-ache, backache, difficulties in getting to sleep and feeling dizzy show disordered thresholds. Similar analyses among individual countries show that disordered thresholds appear in all countries except for Finland: for four items in Denmark, six items in Norway and three items in Sweden. In all of these countries items 2, 3 and 8 show disordered thresholds.

### Rasch-analysis of revised set of eight items with three response categories

**Fig. 3:**
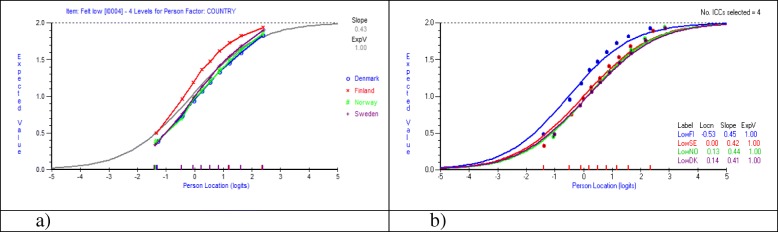
**a**-**b** Differential item functioning across countries for item Feeling Low, before (**a**) and after (**b**) resolving DIF. Set of eight items with three response categories. Entire sample of students 11, 13 and 15 years old

According to the F-values calculated in the ANOVA *Feeling low* was the item with the largest magnitude of DIF. In Fig. 3 a-b expected value curves for item Feeling Low before and after resolution of DIF are shown based on data including all boys and girls 11, 13 and 15 years old.

Figure 3 a-b shows that item Feeling Low shows evidence of DIF, i.e. more than one EVC is required to predict the responses on this item. Figure 3 a-b shows that along the entire latent variable, students in Finland score higher values (=less frequent symptoms) compared to students in the other three countries, given the same location on the latent trait. After resolving the item Feeling Low the DIF is confirmed by different item location values, i.e. uniform DIF.

**Fig. 4:**
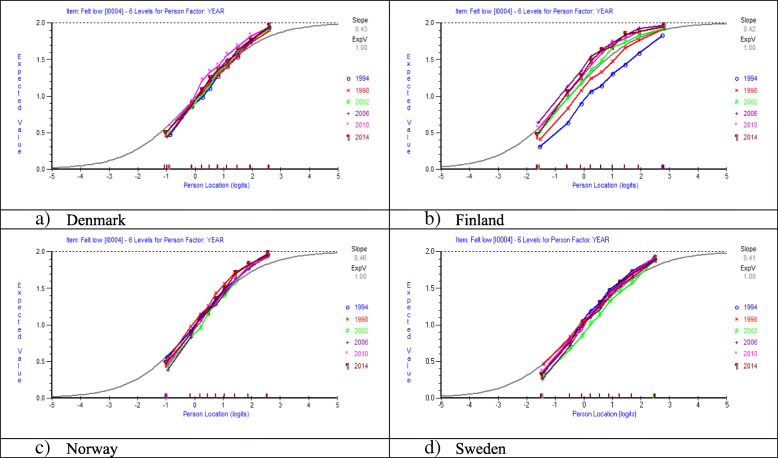
**a-d** Differential Item Functioning across years of investigation for the item Feeling Low in four Nordic countries. Set of eight items with three response categories. Entire sample of students 11, 13 and 15 years old

In Fig. 4 a-d expected value curves for the item Feeling Low across years of investigations in each country are shown including all boys and girls 11, 13 and 15 years old.

Figure 4 a-d shows only small evidence of DIF across years of investigations in Denmark, Norway and Sweden, while in Finland DIF is very obvious. At the first year of investigation in 1994 students in Finland score much lower (=more frequent complaints) than at the last years of investigations in 2010 and 2014, given the same location on the latent trait.

### Trend analyses based on person mean values of psychosomatic symptoms among 15 years old boys and girls

The Person Separation Index which was calculated for four items sets and separately for boys and girls varied between 0.758 and 0.636, indicating good or reasonable power of the fit analyses enabling differentiation of the persons. The independent t-tests based on comparisons of person location values from two different subsets within each item set indicated no evidence of multidimensionality. In all eight tests that were carried out, the proportion of statistically significant t-tests was below the critical value of 5%. Analysis of the correlations between the person-item residuals showed that all the correlations was below 0.3 in all item sets indicating no or only small response dependence.

**Fig. 5:**
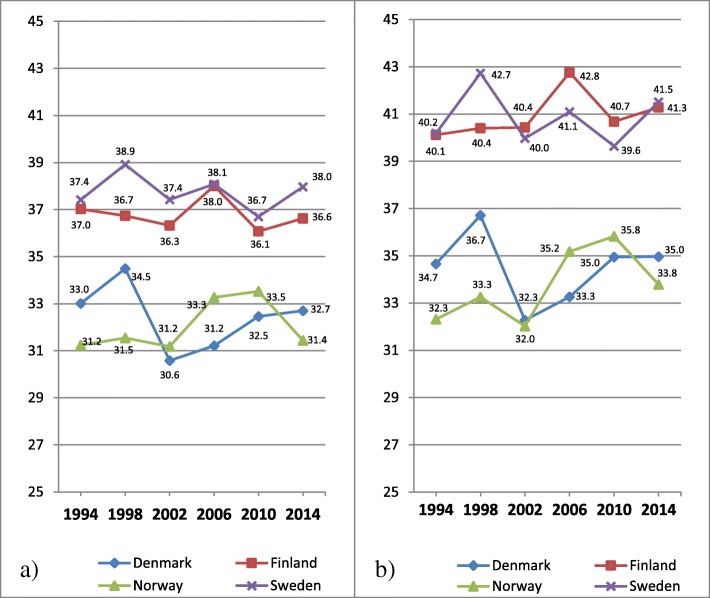
**a-b** Mean values of psychosomatic symptoms on a transformed 0–100 score scale among 15 years old boys, distributed by countries. **a** Original eight items with five response categories; **b** Seven items set with three response categories. The higher the value, the higher degree of psychosomatic symptoms

In Fig. 5 a-b mean values of psychosomatic symptoms among 15 years old boys across years of investigations in each country are shown.

Cross country comparisons based on the original item set shown in Fig. 5 a show that the four Nordic countries are gathered into two main groups: Denmark and Norway with relatively low mean values, and Finland and Sweden with relatively high mean values. Among all countries there are however no or only small differences in mean values, when comparing the first and last years of investigations although there are some fluctuations between individual years. As a whole this pattern holds also for the revised item set shown in Fig. 5 b, although the mean values among three of the countries are slightly higher at the last year of investigation than at the first. The small difference in mean values between Sweden and Finland in 2014 almost disappears in Fig. 5 b.

**Fig. 6:**
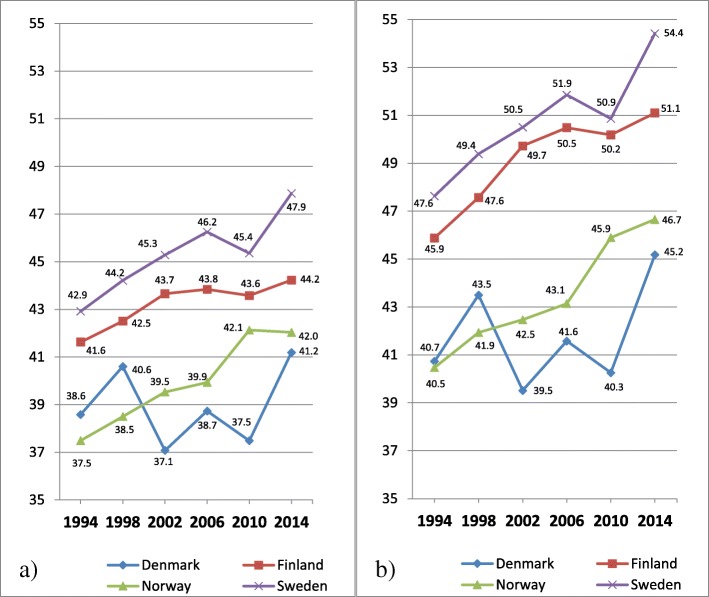
**a-b** Mean values of psychosomatic symptoms on a transformed 0–100 score scale among 15 years old girls, distributed by countries. **a** Original eight items with five response categories; **b** Seven items with three response categories. The higher the value, the higher degree of psychosomatic symptoms

In Fig. 6 a-b mean values of psychosomatic symptoms among 15 years old girls across years of investigations in each country are shown.

Cross country comparisons based on the original item set shown in Fig. 6 a show that the four Nordic countries are more differentiated among girls than shown for boys in Fig. 5 a, with Sweden obviously showing the highest mean values.

In contrast to boys, among all countries the mean values are higher at the last year of investigation than at the first, indicating an increase of psychosomatic symptoms. Except for Denmark, the curves turn upward between each year of investigation. Comparing the first and last years of investigations, the changes are bigger for all countries for the revised item set shown in Fig. 6 b.

### Trend analyses based on cut of value for higher degree of psychosomatic symptoms among 15 years old boys and girls

**Fig. 7:**
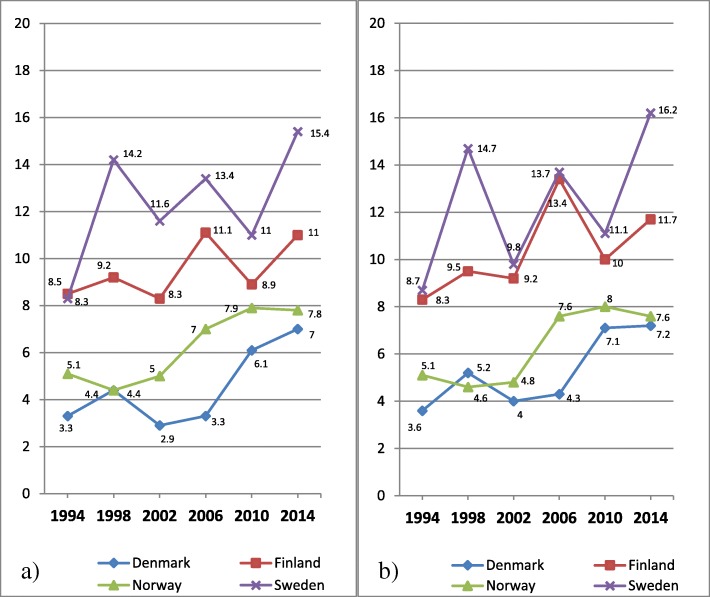
**a-b** Psychosomatic symptoms among 15 years old boys, distributed by countries. **a** Original eight items with five response categories; **b** Seven items set with three response categories. Proportion of students on a transformed 0–100 score scale at or above the 90th percentile (worse health) for the entire sample in 1994

In Fig. 7 a-b, the proportion of 15 years old boys at or above the 90th percentile (=higher degree of psychosomatic symptoms) is shown, across years of investigations and country.

In contrast to Fig. 5 a showing almost no changes of mean values for boys, Fig. 7 a shows an increasing trend of psychosomatic symptoms. The proportion of boys reporting a higher degree of psychosomatic symptoms is bigger at the last year of investigation than at the first in all four countries, with some fluctuations between individual years. Similar to Fig. 5 a showing mean values, the prevalence of higher degree of psychosomatic symptoms among boys is highest in Sweden, followed by Finland, Norway and Denmark. These patterns are about the same in Fig. 7 b based on the revised item set.

**Fig. 8:**
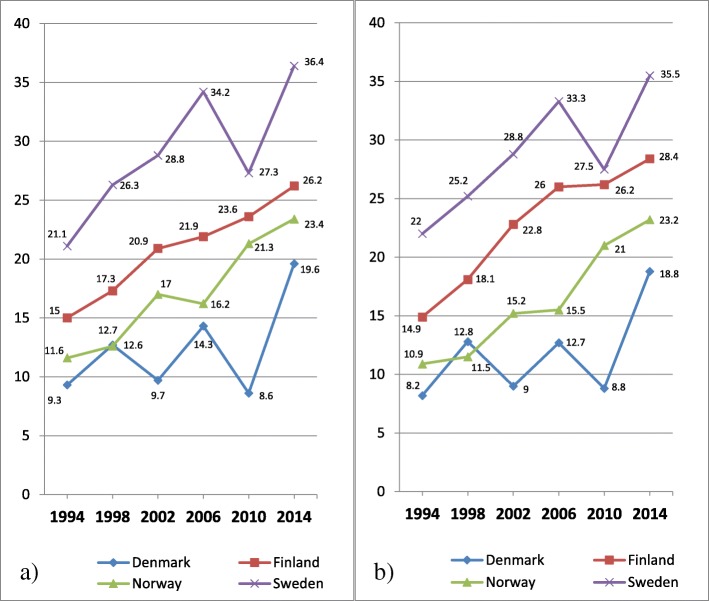
**a-b** Psychosomatic symptoms among 15 years old girls, distributed by countries. **a** Original eight items with five response categories; **b** Seven items with three response categories. Proportion of students on a transformed 0–100 score scale at or above the 90th percentile (worse health) for the entire sample in 1994

In Fig. 8 a-b, the proportion of 15 years old girls at or above the 90th percentile (=higher degree of psychosomatic symptoms) is shown, across years of investigations and country.

Similar to Fig. 6 a showing mean values for girls, Fig. 8 a shows an increasing trend of psychosomatic symptoms. The proportion of girls reporting a higher degree of psychosomatic symptoms is bigger at the last year of investigation than at the first in all four countries, with some fluctuations between individual years.

Similar to the Fig. 6 a showing mean values for girls, the prevalence of higher degree of psychosomatic symptoms is higher in Finland and Sweden than in Denmark and Norway, across all years of investigations. These patterns are about the same in Fig. 8 b based on the revised item set.

In contrast to Fig. 7 a and b for boys, a comparison between Fig. 8 a and b shows that by removal of the item Feeling Low and collapsing two pairs of response categories the comparisons between Finland and Sweden of changes over time are clearly affected. While the original measure of psychosomatic symptoms based 8 items and 5 categories shows an increase of over time with 11% points for Finland and 15% points for Sweden, the measure based on 7 items and 3 response categories shows the same magnitude of increase (13% points) for both Finland and Sweden.

In Table 3 differences in person mean values of psychosomatic symptoms among 15 years old students between four Nordic countries in 1994 are shown for four item sets.

**Table 3:** Differences in person mean values of psychosomatic symptoms on non-transformed scales among 15 years old students between four Nordic countries in 1994

		Denmark				Finland				Norway			
		Set A It 8 5 cat	Set B It 8 3 cat	Set C It 7 5 cat	Set D It 7 3 cat	Set A It 8 5 cat	Set B It 8 3 cat	Set C It 7 5 cat	Set D It 7 3 cat	Set A It 8 5 cat	Set B It 8 3 cat	Set C It 7 5 cat	Set D It 7 3 cat
Finland	Girls	−0.29 (− 0.35 to − 0.23)	− 0.35 (− 0.43 to − 0.26)	−0.32 (− 0.38 to − 0.26)	−0.37 (− 0.46 to − 0.29)								
	Boys	−0.33 (− 0.40 to − 0.27)	−0.42 (− 0.52 to − 0.34)	− 0.29 (− 0.36 to − 0.23)	−0.37 (− 0.46 to − 0.29)								
Norway	Girls	0.10 (0.03 to 0.16)	0.06 (− 0.03 to 0.15)	0.06 (0.00 to 0.12)	0.02 (− 0.07 to 0.11)	0.35 (0.28 to 0.41)	0.37 (0.28 to 0.45)	0.34 (0.28 to 0.40)	0.36 (0.27 to 0.44)				
	Boys	0.14 (0.07 to 0.20)	0.12 (0.03 to 0.20)	0.17 (0.10 to 0.23)	0.15 (0.07 to 0.23)	0.43 (0.36 to 0.50)	0.49 (0.41 to 0.59)	0.42 (0.36 to 0.49)	0.48 (0.40 to 0.58)				
Sweden	Girls	−0.39 (− 0.46 to − 0.32)	− 0.44 (− 0.54 to − 0.35)	− 0.42 (− 0.49 to − 0.36)	−0.47 (− 0.57 to − 0.38)	−0.11 (− 0.18 to − 0.05)	−0.11 (− 0.21 to − 0.02)	−0.13 (− 0.19 to − 0.07)	−0.11 (− 0.21 to − 0.03)	−0.43 (− 0.50 to − 0.36)	−0.45 (− 0.55 to − 0.36)	−0.44 (− 0.51 to − 0.37)	−0.44 (− 0.54 to − 0.35)
	Boys	−0.37 (− 0.44 to − 0.31)	−0.39 (− 0.49 to − 0.31)	−0.35 (− 0.41 to − 0.29)	−0.38 (− 0.47 to − 0.30)	−0.03 (− 0.10 to 0.04)	0.02 (− 0.07 to 0.12)	−0.05 (− 0.12 to 0.01)	−0.01 (− 0.10 to 0.09)	−0.46 (− 0.53 to − 0.40)	−0.47 (− 0.56 to − 0.38)	−0.47 (− 0.54 to − 0.41)	−0.49 (− 0.58 to − 0.40)

Cohen’s d calculated for four different item sets separately for boys and girls. Confidence interval within parentheses. Calculations based on mean values from the original variable

Table 3 confirms with formal statistics the differences in mean values between the four countries at the first year of investigation displayed in Figs. 5 a-b (boys) and 6 a-b (girls). The largest effect sizes are found for comparisons between Denmark vs Finland, Denmark vs Sweden, Finland vs Norway and Norway vs Sweden. Inversely, the smallest effect sizes are found for comparisons between Denmark and Norway, and Finland and Sweden.

In Table 4 differences in person mean values of psychosomatic symptoms among 15 years old students between four Nordic countries in 2014 are shown for four items sets.

**Table 4:** Differences in person mean values of psychosomatic symptoms on non-transformed scales among 15 years old students between four Nordic countries in 2014

		Denmark				Finland				Norway			
		Set A It 8 5 cat	Set B It 8 3 cat	Set C It 7 5 cat	Set D It 7 3 cat	Set A It 8 5 cat	Set B It 8 3 cat	Set C It 7 5 cat	Set D It 7 3 cat	Set A It 8 5 cat	Set B It 8 3 cat	Set C It 7 5 cat	Set D It 7 3 cat
Finland	Girls	−0.24 (−0.29 to − 0.16)	−0.24 (− 0.32 to − 0.14)	−0.32 (− 0.38 to − 0.25)	−0.35 (− 0.42 to − 0.24)								
	Boys	−0.26 (− 0.33 to − 0.18)	−0.31 (− 0.40 to − 0.20)	−0.30 (− 0.36 to − 0.22)	−0.35 (− 0.44 to − 0.25)								
Norway	Girls	−0.06 (− 0.16 to 0.02)	−0.08 (− 0.22 to 0.02)	−0.06 (− 0.16 to 0.01)	−0.08 (− 0.22 to 0.02)	0.17 (0.07 to 0.23)	0.15 (0.02 to 0.23)	0.26 (0.16 to 0.31)	0.26 (0.12 to 0.34)				
	Boys	0.08 (−0.04 to 0.16)	0.05 (−0.09 to 0.16)	0.08 (− 0.03 to 0.16)	0.06 (− 0.08 to 0.17)	0.33 (0.22 to 0.40)	0.35 (0.20 to 0.44)	0.37 (0.26 to 0.43)	0.40 (0.25 to 0.49)				
Sweden	Girls	−0.52 (−0.57 to − 0.45)	−0.53 (− 0.61 to − 0.43)	−0.51 (− 0.56 to − 0.43)	−0.52 (− 0.59 to − 0.42)	−0.30 (− 0.35 to − 0.24)	−0.31 (− 0.38 to − 0.23)	−0.20 (− 0.24 to − 0.14)	−0.19 (− 0.26 to − 0.12)	−0.46 (− 0.50 to − 0.36)	−0.45 (− 0.52 to − 0.31)	−0.45 (− 0.49 to − 0.35)	−0.44 (− 0.50 to − 0.30)
	Boys	−0.35 (− 0.41 to − 0.26)	−0.37 (− 0.45 to − 0.27)	−0.33 (− 0.39 to − 0.25)	−0.35 (− 0.43 to − 0.24)	−0.09 (− 0.15 to − 0.02)	−0.08 (− 0.16 to 0.01)	−0.04 (− 0.10 to 0.02)	−0.01 (− 0.09 to 0.07)	−0.41 (− 0.47 to − 0.30)	−0.41 (− 0.49 to − 0.26)	−0.40 (− 0.46 to − 0.29)	−0.39 (− 0.47 to − 0.25)

Cohen’s d calculated for four different item sets separately for boys and girls. Confidence interval within parentheses. Calculations based on mean values from the original variable

Table 4 confirms with formal statistics the differences in mean values between the four countries at the last year of investigation displayed in Figs. 7 a-b (boys) and 8 a-b (girls). The largest effect sizes are found for comparisons between Denmark vs Finland, Denmark vs Sweden, Finland vs Norway and Norway vs Sweden. Inversely, the smallest effect sizes are found for comparisons between Denmark and Norway, and among boys Finland and Sweden. In contrast to the comparison at the first year of investigation, comparisons at 2014 show that the differences among girls between Finland and Sweden are bigger, in particular for the original items set. While the differences between effect sizes are small between the four different item sets for most comparisons, the discrepancies are bigger for Finland when compared with the other three countries.

## Discussion

The Rasch analysis of the HBSC-SCL on psychosomatic symptoms reveals two major measurement problems that may potentially distort comparisons between countries and across years of investigations: First, the item Feeling low does not work in the same way across countries. The item responses of students with the same location on the latent trait are not the same across all countries, i.e. there is evidence of DIF. Second, some items are showing disordered thresholds which may indicate that the ordering of the categories for some of the eight items does not work as intended. The trend analyses of the data including 15 years old adolescents show that these problems in item functioning do affect some comparisons between countries and across time.

HBSC-data for the Nordic countries have not previously been psychometrically evaluated with a focus on the operating characteristic of the items and invariance across time and countries. As regards DIF, the problems detected in the present study seem to mainly originate from the Finnish data. A closer examination of the Finnish questionnaires shows that the item “feeling low” in the Finnish questionnaire has been translated into “felt depressed”. Since this possible source of the country DIF has been identified, different ways are available to address this problem in future studies. One option could be to remove this specific item, in line with the present study although it may affect the validity negatively as well as decrease the precision of measurement, reflected by the person separation index in the Rasch analysis. Another option could be to resolve the DIF item according to country and using principles of test equating on the resolved item, which would retain precision of measurement but probably still affect validity negatively. Since there are Finnish data available including an item with the intended meaning (Feeling low), a third option would be to replace the mistranslated Finnish item. Further investigations may indicate which operations are preferable in order to bring the data from Finland in better correspondence with data collected in other countries in the HBSC study. Similarly, potential problems in measurement related to the categorisation of the items also need to be addressed.

Given that there are solutions at hand for the identified problems in measurement, the HBSC data have a great potential for solid analyses of time trends among the Nordic countries. Overall, among all four Nordic countries, the proportion of boys and girls with higher degree of psychosomatic symptoms has increased over time. Although that proportion is clearly higher 2014 than 1994 in all countries and among both boys and girls, the shape of most of the trends is nonlinear and there are fluctuations between years of investigations. As a whole, the highest proportion of students with higher degree of psychosomatic symptoms is to be found in Sweden, followed by Finland, Norway and Denmark. Among girls this hierarchy applies to all years of investigations and among boys for all years except for 1994. The HBSC data also enable extensive analyses of possible explanations of the deteriorating mental health among adolescents in the Nordic countries. Since the HBSC data are repeatedly collected every fourth year and cover a long time period, the data are especially suitable for analyses that take societal changes into account.

Not surprisingly, the current study also illustrates how analyses based on a measure of central tendency and a measure of position may reveal different views of the trend patterns if the dispersion of the data changes across time. Among all four Nordic countries, the proportion of 15 years old boys and girls with higher degree of psychosomatic symptoms has increased during the last decades. Among boys, this trend based on a measure of position is not accompanied by changed mean values comparing the first and last years of investigations. In fact, the impact of increasing levels of higher degree of psychosomatic symptoms is cancelled out by a simultaneous increase of lower degree of psychosomatic symptoms. In contrast, among girls there are changes in mean values indicating an increase of psychosomatic symptoms. The results indicate an increasing heterogeneity among boys, which may reflect a widening health gap.

While the trend patterns among boys were not much affected by the problems pertaining to the original measure of psychosomatic symptoms, the comparisons among girls between Finland and Sweden were affected. For example, a 4 percentage point difference between 15 years old girls in Finland and Sweden concerning the rate of increase of psychosomatic symptoms from 1994 to 2014 disappears when the problems with DIF and disordered thresholds are taken into account. Hence, in addition to confirming increasing rates of adolescent mental health problems in the Nordic countries, the substantive analyses in the current study show that Finland is joining Sweden in having the sharpest increase among older adolescents, in particular among girls.

## Conclusions

Some of the cross-country comparisons were distorted because of Differential Item Functioning and problems related to disordering of the item thresholds. The comparisons among girls between Finland and Sweden were affected by the problems pertaining to the original measure of psychosomatic symptoms, while the trend patterns among boys were not much affected. In addition to confirming increasing rates of adolescent mental health problems in the Nordic countries, the substantive analyses in the current study show that Finland is joining Sweden in having the sharpest increase among older adolescents, in particular among girls.

To improve the functioning of the scale the DIF item could be removed or replaced and response categories collapsed in post hoc analyses.

Two additional comments are in order. First, while there are previous studies supporting a unidimensional view of the HBSC-SCL [21, 22], some studies based on confirmatory factor analysis have indicated that the HBSC-SCL may be conceptualised by two highly correlated dimensions, a psychological and a somatic [22, 23].

Second, although the invariant properties of the measurement instrument is critical in cross-country comparisons, also other factors may affect the validity of the health comparisons across countries. Among those, differences between countries in the composition of the samples and variations in participating rates are crucial and may challenge the comparisons across countries and time.

## Abbreviations

ANOVA: Analysis of variance
DIF: Differential Item Functioning
EVC: Expected value curve
HBSC: Health behaviour in school-aged children
PCA: Principal component analysis
RMT: Rasch Measurement Theory
WHO: World Health Organization

## References

[1] Rutter M, Smith DJ (1995). Psychosocial disorders in young people: time trends and their causes.

[2] Bor W, Dean AJ, Najman J, Hayatbakhsh R (2014). Are child and adolescent mental health problems increasing in the 21st century? A systematic review. Aust N Z J Psychiatry.

[3] Collishaw S (2015). Annual research review: secular trends in child and adolescent mental health. J Child Psychol Psychiatry.

[4] Currie C, Zanotti C, Morgan A, Currie D, de Looze M, Roberts C (2012). Social determinants of health and well-being among young people. Health behaviour in school-aged children (HBSC) study: international report from the 2009/2010 survey.

[5] Inchley J, Currie D, Young T, Samdal O, Torsheim T, Augustson L (2016). Growing up unequal: gender and socioeconomic differences in young people’s health and well-being. Health behaviour in school-aged children (HBSC) study: international report from the 2013/2014 survey.

[6] Ottova-Jordan V, Smith OR, Gobina I, Mazur J, Augustine L, Cavallo F (2015). Trends in multiple recurrent health complaints in 15-year-olds in 35 countries in Europe, North America and Israel from 1994 to 2010. Eur J Pub Health.

[7] Potrebny T, Wiium N, Lundegard MMI. Temporal trends in adolescents' self-reported psychosomatic health complaints from 1980-2016: a systematic review and meta-analysis. PLoS One. 2017;12(11). 10.1371/journal.pone.0188374 PMC5705135 29182644

[8] Verhulst F (2015). Commentary: physical health outcomes and health care have improved so much, so why is child mental health getting worse? Or is it? A commentary on Collishaw (2015). J Child Psychol Psychiatry.

[9] Hagquist C, Välimaa R, Simonsen N, Suominen S (2017). Differential item functioning in trend analyses of adolescent mental health – illustrative examples using HBSC-data from Finland. Child Indic Res.

[10] Ravens-Sieberer U, Erhart M, Torsheim T, Hetland J, Freeman J, Danielson M (2008). An international scoring system for self-reported health complaints in adolescents. Eur J Pub Health.

[11] Masters GN (1982). A Rasch model for partial credit scoring. Psychometrika.

[12] Hagquist C, Bruce M, Gustavsson JP (2009). Using the Rasch model in nursing research: an introduction and illustrative example. Int J Nurs Stud.

[13] Andrich D (1988). Rasch models for measurement. Report no.: 68, Quantitative Applications in the social sciences.

[14] Andrich D, Everitt BS, Howell DC (2005). Rasch models for ordered response categories. Encyclopedia of Statistics in Behavioral Science.

[15] Andrich D, de Jong JHAL, Sheridan BE, Rost J, Langeheine R (1997). Diagnostic opportunities with the Rasch model for ordered response categories. Applications of latent trait and latent class models in the social sciences. Münster and New York: Waxmann Verlag GMBH.

[16] Andrich D, Hagquist C (2012). Real and artificial differential item functioning. J Educ Behav Stat.

[17] Andrich D, Hagquist C (2015). Real and artificial differential item functioning in polytomous items. Educ Psychol Meas.

[18] Hagquist C, Andrich D (2015). Determinants of artificial DIF – a study based on simulated polytomous data. Psychol Test Assess Model.

[19] Hagquist C, Andrich D (2017). Recent advances in analysis of differential item functioning in health research using the Rasch model. Health Qual Life Outcomes.

[20] Andrich D, Sheridan B, Luo G (2014). RUMM2030: A windows interactive program for analysing item response data with Rasch Unidimensional Models for Measurement.

[21] Hagquist C, Andrich D (2004). Measuring subjective health among adolescents in Sweden: a Rasch analysis of the HBSC-instrument. Soc Indic Res.

[22] Haugland S, Wold B, Stevenson JIM, Aaroe LE, Woynarowska B (2001). Subjective health complaints in adolescence: a cross-national comparison of prevalence and dimensionality. Eur J Pub Health.

[23] Hetland J, Torsheim T, Aarø LE (2002). Subjective health complaints in adolescence: dimensional structure and variation across gender and age. Scand J Public Health.

